# Early Outcomes of Out-of-Hospital Cardiac Arrest after Early Defibrillation: a 24 Months Retrospective Analysis

**Published:** 2006-10-01

**Authors:** Paolo Terranova, Paolo Valli, Barbara Severgnini, Simonetta Dell'Orto, Greco Enrico Maria

**Affiliations:** 1 Divisione e Cattedra di Cardiologia, Dipartimento di Medicina, Chirurgia e Odontoiatria dell'Universita degli Studi di Milano, Azienda Ospedaliera "S. Paolo" - Polo Universitario, Milano; 2 U.O. Cardiologia, Presidio Ospedaliero "Causa Pia Uboldo", Cernusco S/N, AOSP Melegnano, Milano

**Keywords:** out-of-hospital cardiac arrest, early defibrillation, automated external defibrillators

## Abstract

**Introduction:**

Cardiovascular disease remains the most common cause of death in the United States and most other Western nations. Among these deaths, sudden, out-of-hospital cardiac arrest claims approximately 1000 lives each day in the United States alone. Most of these cardiac arrests are due to ventricular fibrillation. Though highly reversible with the rapid application of a defibrillator, ventricular fibrillation is otherwise fatal within minutes, even when cardiopulmonary resuscitation is provided immediately. The overall survival rate in the United States is estimated to be less than 5 percent. Recent developments in automated-external-defibrillator technology have provided a means of increasing the rate of prompt defibrillation after out-of-hospital cardiac arrest. After minimal training, nonmedical personnel (e.g., flight attendants and casino workers) are also able to use defibrillators in the workplace, with lifesaving effects. Nonetheless, such programs have involved designated personnel whose job description includes assisting persons who have had sudden cardiac arrest. Data are still lacking on the success of programs in which automated external defibrillators have been installed in public places to be used by persons who have no specific training or duty to act.

**Materials and Methods:**

All patients who had an out-of-hospital cardiac arrest between January 2003 and December 2004 and who received early defibrillation for ventricular fibrillation were included. We conducted a 24 months retrospective population-based analysis of the outcome in our population.

**Results:**

Over a 24 month period, 446 people had non-traumatic cardiac arrest, and in all of them it was observed to be ventricular fibrillation. In a very few cases, the defibrillator operators were good Samaritans, acting voluntarily. Eighty-nine patients  (about 19%) with ventricular fibrillation were successfully resuscitated, including eighteen who regained consciousness before hospital admission.

**Conclusion:**

Automated external defibrillators deployed in readily accessible, well-marked areas, are really very effective in assisting patients with cardiac arrest. However, it's quite true that, in the cases of survivors, most of our users had good prior training in the use of these devices.

## Introduction

Survival after out-of-hospital cardiac arrest depends on a sequence of events termed "the chain of survival" [1-3], which involves rapid access to emergency medical care, cardiopulmonary resuscitation (CPR), defibrillation, and advanced care [3]. Several studies have shown that rapid defibrillation after an out-of-hospital cardiac arrest with ventricular fibrillation is the single most important determinant of outcome [4-10]. In most places, survival rates range from 3 to 10 percent because the chain of survival is not promptly implemented, although with the increasing availability of early defibrillation the rates are improving [4-10]. The principle of early defibrillation, strongly endorsed by the 1992 National Conference on Cardiopulmonary Resuscitation and Emergency Care [14], supports the use of this intervention by emergency personnel who are first at the scene. In the city of Rochester in Olmsted County, Minnesota, early defibrillation by police was implemented in late 1990. The rate of survival to hospital discharge with the use of this defibrillation program is 40 percent [6,7].

There is a paucity of data about long-term survival and quality of life of survivors of out-of-hospital cardiac arrest. In Rochester and Olmsted County, survivors of ventricular fibrillation receive subsequent treatment and follow-up at one institution, a factor that facilitates long-term follow-up of this closed population. We conducted this study to determine the effect of rapid defibrillation and aggressive care on early survival after defibrillation until at hospital admission.

## Material and Methods

All patients who had an out-of-hospital cardiac arrest with ventricular fibrillation between January 2003 and December 2004 and who received defibrillation from medical personnel in Cernusco Sul Naviglio Hospital Emergency Room (Milan, Italy) were included. Data regarding the cardiac arrest and subsequent outcomes were collected in a prospective manner and re-analyzed from an historical point of view.

Few data have been previously reported by other groups4-7. Trained personnel, primarily police officers and, in some cases, firefighters, provided defibrillation using automated external defibrillators. Paramedics provided advanced life support. In this emergency medical system, telephone calls to 118 are received at a public-safety communications center, which then dispatches ambulance, police and firefighters. The interval from the 118 call to the administration of the first shock was determined by synchronizing the defibrillator time obtained from the automated external defibrillator itself with the dispatch time recorded at the communications center [4-7].

Emergency medical personnel confirmed that a patient was pulseless and then attached an automated external defibrillator; at that time CPR, if ongoing, was discontinued so as not to interfere with the device. Return of spontaneous circulation was considered to have occurred if the initial shocks restored circulation and subsequently maintained it. No epinephrine or other vasoactive drug was needed in this group of patients. Patients who required advanced life support in the absence of the return of spontaneous circulation received epinephrine, along with other drug therapy if needed, and underwent endotracheal intubation. All patients were transported to a single hospital. The underlying cause of the cardiac arrest was established and categorized as myocardial infarction or acute coronary syndrome on the basis of electrocardiographic evidence of ST-segment elevation or angiographic evidence of acute occlusion, ischemic coronary heart disease without obvious acute coronary syndrome or myocardial infarction as previously defined, non ischemic heart disease, or some other cardiac cause with no obvious organic heart disease. The outcome in the hospital emergency department was evaluated (Table 1).

Survival was defined by an overall performance category score [15-18] of 1 (good overall capability) or 2 (moderate overall disability - patient is conscious and performs independent activities of daily life but has moderate cerebral or noncerebral organ-system dysfunction) at hospital emergency room discharge. Patients who died in the hospital (score of 5) or who had a score of 3 (severe overall disability - patient is dependent on others for daily support and has severe cerebral or noncerebral organ-system dysfunction) or 4 (coma) at the time of discharge were considered nonsurvivors. We ascertained the patients' vital status by reviewing our patient-data registry.

We reported our data using descriptive statistical models. No statistical method was used to estimate survival rates, e.g. the Kaplan-Meier product-limit one, because it was not a purpose of this observational and retrospective study. Moreover, we did not determine expected survival by calculating survival rates during the considered period in our control population

## Results

**Figure 1:**
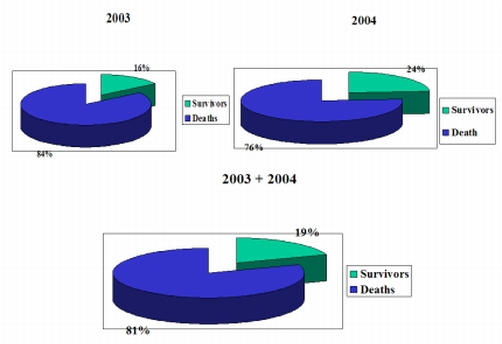
Percentages of survivors after BLS/ACLS and Early Defibrillation

During the study period (January 2003 - December 2004), 446 subjects experienced an out-of-hospital non-traumatic cardiac arrest of presumed or documented cardiac origin. All patients of our database presented in ventricular fibrillation or asystole. Details of the initial resuscitation before hospitalization have been reported previously, being the same used in formerly described trials [4-7]. Of the patients with ventricular fibrillation, eighty-nine patients  (about 19%) restored a spontaneous circulation after defibrillation and were admitted to the emergency department (Figure 1). Table 1 shows the main clinical data and the outcome after admission to the hospital emergency room.  The mean time from the 118 call to the administration of the first shock was 15.7 ± 4.4 minutes (range, 5.5 to 26.4) among survivors and 18.2 ± 4.7minutes (range, 13.4 to 32.4) among nonsurvivors (P=0.024). Bystanders performed CPR on 214 patients (48 percent). The performance of CPR was not assessed in terms of its apparent effectiveness. Unfortunately, we have no chance of having data about a probable interesting significant effect of CPR prior to the shock. It would have been very interesting to know how much time elapsed between the arrest and the call and the correlation with bystander CPR to get a more complete picture, but this data is not currently available because of the recording system of our emergency care organization.

A very few of our resuscitated patients (15 subjects on 89, 16.8%) underwent echocardiography during their emergency room experience after the out-of-hospital cardiac arrest. The average ejection fraction in this group was 0.42 ± 0.18 at base line. Among the survivors, all patients with a reversible cause of the out-of-hospital cardiac arrest - acute myocardial ischemia - were successfully treated. Ventricular-fibrillation arrest was considered part of this event in all these 89 patients, and they were all candidates for antiarrhythmic drug therapy.

Among patients who were considered candidates for antiarrhythmic therapy, 35 patients received amiodarone (600 mg daily for a fortnight and then 200 mg per day) while 54 subjects received sotalol (80 mg three times a day). None of our patients received an implantable cardioverter-defibrillator, because arrhythmic events, occurring during a myocardial ischemia, were considered triggered by this ischemic event.

## Discussion

In this population-based retrospective study on the outcomes of patients during a program of rapid defibrillation, subjects who experienced out-of-hospital cardiac arrest and survived to hospital emergency room admission and discharge, had probably a long-term survival rate that may be considered equivalent to that of age-, sex-, and disease-matched patients who did not experience it. A nearly normal quality of life and return to work were reported by the majority of survivors discharged by our Hospital.

These data serve as a benchmark and illustrate what can be achieved in a community setting with an aggressively implemented program of early defibrillation. As previously reported [4-7], survival after ventricular fibrillation in this cohort was relatively high (about 19%). Previous studies of patients with an out-of-hospital cardiac arrest with ventricular fibrillation have reported survival rates after hospital discharge ranging from 3 to 33 percent in a variety of settings [8-13].  In communities without access to early defibrillation, mortality rates exceed 90 to 95 percent [12,13].

In contrast, in communities that have programs of early defibrillation, survival rates of 15 to 40 percent have been reported [4-7,11,19,20]. In Rochester, Minnesota, during a four-year historical-control period before the implementation of the early-defibrillation program in 1990, the rate of survival to discharge was 28 percent. In the current study, the rate was 19 percent after the program was implemented. This value represents a marked percent increase in survival as compared with reported outcomes in other locations [8-13,19,20] and is most likely due, at least in part, to the relatively short interval between the 118 call and the administration of the first shock and to aggressive early management in the hospital.

It is likely to suppose that the use of revascularization procedures of culprit lesions and antiarrhythmic therapy started in the hospital, coupled with the higher survival rate associated with early defibrillation, would increase the long-term survival rate.

Many factors may have predisposed the survivors to an increased risk of subsequent death. First, this population had already experienced a ventricular-fibrillation-induced cardiac arrest, and this fact has been widely described as being an important predictor [21,22]. For example, the five-year mortality rate among patients with a history of cardiac arrest in the Cardiac Arrest Study Hamburg was 36 percent in the group that received an implantable cardioverter-defibrillator and 44 percent in the group that received antiarrhythmic therapy [22]. In a subgroup analysis of 98 patients in the Canadian Implantable Defibrillator Study [23] who presented with out-of-hospital cardiac arrest, the total mortality rate was 18.4 percent at two years and 33.4 percent at five years. Second, although the rate of in-hospital death from cardiac causes was low, recognized predictors of adverse outcomes (multivessel coronary heart disease, diabetes, left ventricular dysfunction, and congestive heart failure) [23] were prevalent among this cohort. Furthermore, the high prevalence of death from noncardiac causes in this study reflects a population at high risk for death as a result of other coexisting diseases.

Previous studies assessing the quality of life in these patients were small, focused on short-term recovery rates (at 3 to 12 months), or used only a subjective assessment tool with broad categories, such as the cerebral performance scale [24-31].

When the standardized SF-36 was used [32], results indicated that the majority of patients had subsequently a nearly normal quality of life similar to that of the general population, with the exception of the degree of vitality. The difference between groups was moderate; vitality scores in the cohort were within 1 SD of U.S. norms. Also, vitality, as measured by the SF-36, may improve with rehabilitation among patients with cardiac disease [33].

These findings in a closed little population validate the results of few previous open studies, which reported an acceptable quality of life after emergency room discharge [29-31,33,34]. In addition, the majority of our patients returned to work (about 65 percent of those who were less than 65 years of age), thus confirming and expanding previous reports that many survivors of out-of-hospital cardiac arrest return to their previous occupation within six months [29-31].

Our study has however several limitations. First, the results were derived from a little population. Our rapid-response program could be difficult to replicate in large cities with skyscrapers. Nevertheless, in such settings, these outcomes might be achieved through the placement of automated external defibrillators on several floors of high-rise structures and the training of security officers in their use. Second, only patients who presented with ventricular fibrillation were included. This was by design, since we chose to assess the effect of rapid defibrillation on survival and subsequent quality of life at the discharge from an hospital emergency department. However, since the overall rate of death from cardiac causes after discharge was low, these data overall still provide substantial insight into the quality of life of patients with underlying cardiac disease. Third, the results with respect to the quality of life are collective and may not be applicable to individual patients.

Finally, the use of CPR by bystanders in the system we studied has not affected the rate of survival after out-of-hospital cardiac arrest with ventricular fibrillation [4-6]. Previously, it has been reported by Bunch et al [6] that there was no significant difference in the rates of bystander administered CPR between patients who survived out-of-hospital ventricular fibrillation and those who did not survive (42 percent vs. 58 percent, P=0.74). This findings have been substantially replicated in the current study. This finding may reflect the relatively short interval between the 118 call and the administration of the first shock and should therefore not be misinterpreted to apply to other settings involving longer intervals before defibrillation can be attempted.

In summary, the rate of survival to hospital discharge was relatively high in a small city that had a program of rapid defibrillation. The majority of survivors returned to work, and their quality of life was in most respects indistinguishable from that of the general population. The long-term survival rate was relatively similar to that of age-, sex-, and disease-matched controls who did not have an out-of-hospital cardiac arrest.

## Figures and Tables

**Table 1:**
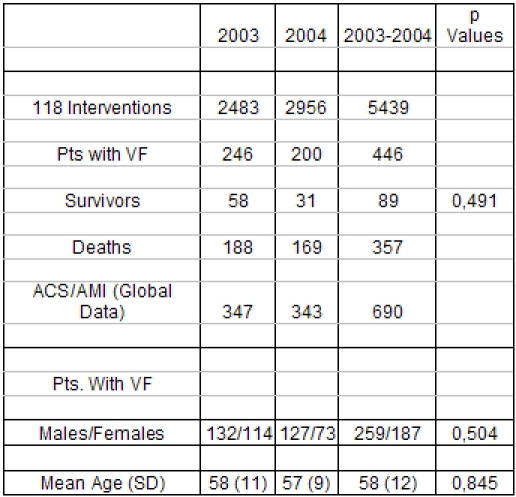
Patients' Main Characteristics (Pts., patients; VF, Ventricular Fibrillation; ACS, Acute Coronary Syndrome; AMI, Acute Myocardial Infarction; SD, standard deviation)
